# Re‐evaluating age‐related attention‐deficit/hyperactivity disorder (ADHD) symptom trajectories using the Japanese ADHD Rating Scale‐5

**DOI:** 10.1002/pcn5.70301

**Published:** 2026-02-17

**Authors:** Ryotaro Shimomura, Yukie Tateno, Keisuke Oyatani, Kotaro Nanba, Eri Shiraishi, Masaru Tateno

**Affiliations:** ^1^ Tokiwa Child Development Center Tokiwa Hospital Sapporo Hokkaido Japan; ^2^ Department of Pediatric Cardiology Fukuoka Children's Hospital Fukuoka Japan; ^3^ Department of Neuropsychiatry Sapporo Medical University School of Medicine Sapporo Hokkaido Japan

**Keywords:** attention‐deficit/hyperactivity disorder, functional impairment, medication, neurodevelopmental disorder, pharmacotherapy, rating scale

## Abstract

**Aim:**

Attention‐deficit/hyperactivity disorder (ADHD) is a neurodevelopmental disorder characterized by inattention (IA), hyperactivity, and impulsivity. In 2022, the Japanese version of the ADHD Rating Scale‐5 (ADHD‐RS‐5) was released based on DSM‐5. Although there are several changes compared to the previous version, few clinical studies have been conducted using the ADHD‐RS‐5. This study aimed to re‐evaluate ADHD‐related characteristics by age group in pediatric psychiatric outpatients, using the ADHD‐RS‐5 regardless of diagnosis, and compare these findings with previous research.

**Methods:**

Participants were all patients aged 5–17 years who visited the Psychiatry Clinic of Tokiwa Hospital or the Tokiwa Child Development Center (Child Psychiatry Clinic) during the study period. Of 503 children, 452 met the inclusion criteria. Primary caregivers completed the ADHD‐RS‐5. Symptom and functional impairment scores were compared by ADHD diagnosis and age group. In the ADHD group, scores were also compared based on pharmacological treatment.

**Results:**

IA and hyperactivity–impulsivity (HI) scores were significantly higher in the ADHD group. Functional impairment scores did not differ significantly in some age groups. HI scores were lower in older age bands, whereas IA scores did not differ significantly across age groups; the cross‐sectional patterns are compatible with relative stability of inattentive symptoms but do not establish longitudinal persistence. Pharmacological treatment was not linked to symptom scores but was associated with higher impairment scores.

**Conclusion:**

Our findings using the ADHD‐RS‐5 showed results consistent with previous reports regarding age‐specific symptom characteristics of ADHD, while also providing new insights into treatment options for ADHD. Clinicians may consider both symptom severity and functional impact when initiating pharmacotherapy. Given medication approval from age 6 in Japan, accurate assessment and diagnosis remain essential, and the ADHD‐RS‐5 may support decision‐making when interpreted alongside clinical judgment and multi‐informant input.

## INTRODUCTION

Attention‐deficit/hyperactivity disorder (ADHD) is a neurodevelopmental disorder characterized by inattention (IA), hyperactivity, and impulsivity. According to the Diagnostic and Statistical Manual of Mental Disorders, Fifth Edition, Text Revision (DSM‐5‐TR), ADHD is diagnosed based on persistent patterns of IA and/or hyperactivity–impulsivity (HI) that interfere with functioning and development.^1^ Due to persistent difficulties associated with IA at home and school, as well as frequent reprimands, individuals with ADHD are often prone to developing negative self‐perceptions, low self‐esteem, anger, and resentment. These emotional responses may contribute to secondary conditions such as school refusal, depression, and anxiety. Impulsivity in ADHD has also been associated with emotional dysregulation, interpersonal difficulties, and various forms of addiction. Recently, attention has increased to its potential links with internet addiction and gaming disorder.^2^

In terms of treatment, Japanese clinical guidelines recommend starting with environmental adjustments and psychosocial interventions. If these are insufficient, pharmacotherapy may be considered.^3^ In more severe cases, the observation period may be shortened, and pharmacotherapy with ADHD medication can be introduced earlier depending on the level of functional impairment. Shared decision‐making is emphasized when selecting treatment options.^4^ However, a recent report suggested an association between initiating ADHD medication and suicidal ideation, requiring clinicians to exercise caution when considering the initiation of ADHD medication.^5^

To ensure accurate diagnosis, ADHD symptoms must be present across multiple settings. Therefore, in addition to direct observation and parental interviews, the use of standardized rating scales can improve diagnostic reliability. In Japan, the ADHD Rating Scale‐IV (ADHD‐RS‐IV), based on DSM‐IV, has been widely used.^6^ It includes 18 items corresponding to the DSM‐IV diagnostic criteria, rated by primary caregivers—mainly parents—or school teachers, to capture symptoms across multiple settings such as home and school. Although the DSM was updated to the fifth edition in 2013, followed by a text revision in 2022, the ADHD‐RS‐IV continued to be widely used in Japan for many years.^1^, ^7^ In 2016, DuPaul et al. published a revised version in accordance with DSM‐5, and in December 2022, the Japanese version of the ADHD Rating Scale for Children and Adolescents (ADHD‐RS‐5) was released.^8^, ^9^ This new version demonstrated good internal consistency, test–retest reliability, construct validity, and criterion‐related validity.^10^

Although the item content remains largely unchanged, several notable updates were introduced: (1) The IA and HI items, previously mixed, are now separated into distinct subscales (symptom scores); (2) Age‐specific forms for children and adolescents were created, including developmentally appropriate behavioral examples; (3) A new scale was added to assess executive function deficits (functional impairment scores). However, few clinical studies have been conducted in Japan using the ADHD‐RS‐5, and it remains largely unclear what utility the revised version offers in ADHD clinical practice.

In this study, we conducted a cross‐sectional analysis using the ADHD‐RS‐5 to re‐evaluate the clinical characteristics of ADHD.

## METHODS

### Participants

Primary caregivers of children aged 5–17 years who visited the Psychiatry Clinic of Tokiwa Hospital or the Tokiwa Child Development Center (Child Psychiatry Clinic) between March 17 and April 5, 2024, were recruited for this study. The attending physician of each child provided a thorough explanation to the caregiver regarding the purpose and procedures of the study, emphasizing that participation was entirely voluntary and that refusal to participate would not result in any disadvantage. Following this explanation, informed consent for participation was requested. Participants were excluded if they met any of the following conditions: lack of informed consent, incomplete responses to any questionnaire, presence of moderate to severe intellectual disability (ID) (IQ < 50 on standardized intelligence testing, or diagnosis clearly documented in medical records), or diagnosis of schizophrenia.

This study was conducted exclusively with follow‐up patients. Therefore, all study participants were patients continuing treatment at Tokiwa Hospital and Tokiwa Child Development Center.

### Diagnostic procedures and clinical team

At the time of the study, the outpatient clinic was staffed by 10 physicians who had more than 5 years of experience in child psychiatry. The clinical team included board‐certified child psychiatrists, board‐certified pediatricians, board‐certified psychiatrists, and physicians holding Autism Diagnostic Observation Schedule, Second Edition (ADOS‐2) clinical and/or research certification.

Diagnosis of neurodevelopmental disorders based on DSM‐5 criteria was established through comprehensive clinical evaluations, including direct observation, structured parental interviews, and standardized psychological assessments when indicated, such as the Parent‐interview autism spectrum disorder (ASD) Rating Scale‐Text Revision (PARS‐TR), ADHD‐RS‐5, and the ADOS‐2, which is considered the gold standard for ASD diagnosis. At Tokiwa Child Development Center, PARS‐TR is routinely administered to all patients at their initial visit. ADOS‐2 and ADHD‐RS‐5 are administered when deemed clinically necessary by the treating physician.

### Procedure

Participants were asked to complete a study questionnaire, which included the ADHD‐RS‐5, with additional items for demographics. The questionnaire collected demographic information about the child, such as age, sex, ADHD diagnosis status, and current use of ADHD medication. The authors verified the accuracy of the reported diagnoses by cross‐referencing the questionnaire responses with medical records. The ADHD‐RS‐5 is a questionnaire used to assess the severity of ADHD symptoms, completed by the child's primary caregiver or school teacher. According to the DSM‐5 diagnostic criteria for ADHD, the scale consists of a total of 18 items—9 for IA and 9 for HI. In the ADHD‐RS‐5, these nine IA items and nine HI items are rated using a 4‐point Likert scale: 0 = Never or Rarely, 1 = Sometimes, 2 = Often, and 3 = Very Often. Accordingly, the total score ranges up to a maximum of 54 points.

### Study design and statistical analysis

Participants were classified into ADHD and non‐ADHD groups. ADHD‐RS‐5 symptom and functional impairment scores were compared (1) between the ADHD and non‐ADHD groups within each age category (5–7, 8–10, 11–13, and 14–17 years), (2) between the ADHD‐only diagnosis group and the ASD comorbidity group, (3) across age categories within each diagnostic group, and (4) between ADHD participants with and without ongoing psychopharmacological treatment for ADHD. Comparisons by ADHD diagnosis, ASD comorbidity, and treatment status were performed using the Mann–Whitney *U* test, whereas comparisons across age categories were conducted using the Kruskal–Wallis test followed by the Steel–Dwass multiple comparison procedure. All statistical analyses were conducted using EZR (version 1.63).^11^ Results are expressed as median [interquartile range], and a *p*‐value < 0.05 was considered statistically significant.

### Ethical considerations

This study was approved by the Ethics Committee of Tokiwa Hospital (TH‐20240115). Written informed consent was obtained from all participants.

## RESULTS

### Demographic and clinical characteristics of the children

**Figure 1:**
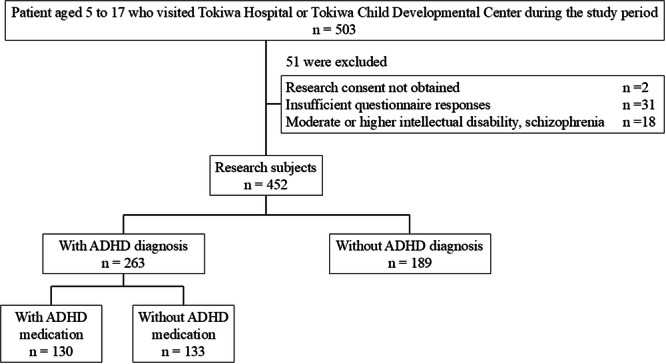
Flow chart for selection of the study subjects. ADHD, attention‐deficit/hyperactivity disorder.

The demographic and clinical characteristics of the children evaluated in this study are presented in Figure 1 and Table 1. Among the 452 children assessed in this study, 263 (58.2%) had been diagnosed with ADHD. There were no significant differences between the ADHD and non‐ADHD groups in mean age, comorbidity rates of ASD or ID, or Full‐Scale Intelligence Quotient (FSIQ) scores on the Wechsler Intelligence Scale for Children‐Fourth Edition (WISC‐IV). However, the proportion of males was significantly higher in the ADHD group. The primary diagnoses in the non‐ADHD group were the following: 99 with ASD (28 of whom also had ID), 17 with stress related disorders (including reactive attachment disorder and adjustment disorder), 16 with anxiety disorders (including social anxiety disorder, generalized anxiety disorder, and selective mutism), 14 with mood disorders (including depression, depressive episodes, bipolar disorder, and mood dysregulation disorder), 11 were diagnosed with mild ID, 5 had other neurodevelopmental disorders (including specific learning disorder and tic disorder), 5 had neurotic disorders (including somatic symptom disorder, conversion disorder, and dissociative disorders), and 2 had eating disorders. In total, 20 had no clear medical diagnosis (e.g., those with neurodevelopmental traits below diagnostic thresholds, or follow‐up outpatient cases where symptoms had already improved). Of the children diagnosed with ADHD, 130 (49.4%) were being treated with ADHD medication. Details of medication treatment: 49 were on guanfacine (GXR), 44 on methylphenidate (MPH), 16 on atomoxetine (ATX), 14 on GXR and MPH combination therapy, 6 on GXR and ATX combination therapy, and 1 on lisdexamfetamine.

**Table 1:** Demographic characteristics of the evaluator's child.

	All *n* = 452	ADHD *n* = 263	Non‐ADHD *n* = 189	*p*‐value
Demographic characteristics				
Age; mean (SD)	10.7 (3.4)	10.8 (3.3)	10.7 (3.7)	0.721
Male, *n* (%)	334 (70.8)	204 (77.5)	116 (61.2)	<0.001*
With ASD, *n* (%)	256 (56.6)	157 (60.0)	99 (52.4)	0.125
With ID, *n* (%)	88 (19.5)	49 (18.6)	39 (20.6)	0.631
Comparison by the WISC‐IV				
Within 2 years, *n* (%)	351 (77.7)	216 (82.1)	135 (71.4)	0.008*
FSIQ; mean (SD)	87.3 (15.3)	88.2 (15.4)	85.9 (15.0)	0.168
ADHD medication, *n* (%)		130 (49.4)		

*Note*: We compared categorical variables using Fisher's exact test and continuous variables using Student's *t*‐test. The results are expressed as means (SD) or numbers (%). Asterisks indicate statistical significance (*p < 0.05).

Abbreviations: ADHD, attention‐deficit/hyperactivity disorder; ASD, autism spectrum disorder; FSIQ, Full‐Scale Intelligence Quotient; ID, intellectual disability; SD, standard deviation; WISC‐IV, Wechsler Intelligence Scale for Children‐Fourth edition.

### Study 1: Comparison by ADHD diagnosis

The results are shown in Table 2. Symptom scores for both IA and HI were significantly higher in the ADHD group than in the non‐ADHD group across all age groups. Functional impairment scores for both IA and HI were also significantly higher in the ADHD group, except for HI scores in the 11–13 age group, where no significant difference was observed.

**Table 2:** Comparison of the symptom scores and impairment scores between presence or absence of the attention‐deficit/hyperactivity disorder (ADHD) diagnosis.

	ADHD	Non‐ADHD	*p*‐value
*Symptom scores*			
All	*n* = 263	*n* = 189	
IA	13.0 (9.0–18.0)	8.0 (4.0–12.0)	<0.001*
HI	7.0 (4.0–11.0)	2.0 (1.0–5.0)	<0.001*
Aged 5–7	*n* = 51	*n* = 49	
IA	13.0 (9.5–16.5)	10.0 (7.0–15.0)	0.023*
HI	9.0 (6.5–14.5)	6.0 (2.0–7.0)	<0.001*
Aged 8–10	*n* = 78	*n* = 44	
IA	14.5 (10.0–18.8)	7.0 (5.0–11.0)	<0.001*
HI	9.5 (6.0–14.0)	2.5 (1.0–5.0)	<0.001*
Aged 11–13	*n* = 71	*n* = 39	
IA	14.0 (9.0–17.0)	8.0 (2.5–11.5)	<0.001*
HI	6.0 (3.5–9.0)	2.0 (0.5–5.0)	<0.001*
Aged 14–17	*n* = 63	*n* = 57	
IA	12.0 (8.0–18.0)	6.0 (3.0–10.0)	<0.001*
HI	5.0 (1.0–9.0)	1.0 (0–3.0)	<0.001*
*Functional impairment scores*			
All	*n* = 263	*n* = 189	
IA	6.0 (3.0–9.0)	3.0 (1.0–7.0)	<0.001*
HI	5.0 (2.0–9.0)	2.0 (0–6.0)	<0.001*
Aged 5–7	*n* = 51	*n* = 49	
IA	4.0 (2.0–6.5)	3.0 (1.0–6.0)	0.049*
HI	4.0 (2.0–6.0)	2.0 (1.0–5.0)	0.023*
Aged 8–10	*n* = 78	*n* = 44	
IA	6.5 (4.0–9.8)	3.0 (1.8–7.0)	<0.001*
HI	6.0 (3.0–9.0)	1.5 (0–6.0)	<0.001*
Aged 11–13	*n* = 71	*n* = 39	
IA	7.0 (5.0–10.0)	5.0 (1.0–7.0)	<0.001*
HI	5.0 (1.5–9.0)	5.0 (0.5–6.5)	0.140
Aged 14–17	*n* = 63	*n* = 57	
IA	7.0 (4.0–9.5)	3.0 (1.0–6.0)	<0.001*
HI	6.0 (2.0–9.0)	1.0 (0–4.0)	<0.001*

*Note*: The results are expressed as medians (IQR; interquartile ranges). The comparisons were calculated using the Mann–Whitney *U* test. Asterisks indicate statistical significance (*p < 0.05).

Abbreviations: HI, hyperactivity–impulsivity; IA, inattention.

### Study 2: Comparison by presence of ASD comorbidity

The results are presented in Table 3. Symptom scores for both IA and HI tended to be higher in the ASD comorbidity group, but no significant differences were found in either comparison.

**Table 3:** Comparison of the symptom and impairment scores between presence or absence of the autism spectrum disorder (ASD) comorbidity.

	With ASD *n* = 157	Without ASD *n* = 106	*p*‐value
IA			
Symptom score	14.0 (10.0–18.0)	12.5 (8.0–17.0)	0.116
Impairment score	7.0 (4.0–10.0)	6.0 (3.0–9.0)	0.0903
HI			
Symptom score	8.0 (4.0–12.0)	7.0 (3.0–11.0)	0.337
Impairment score	5.0 (3.0–9.0)	5.5 (2.0–8.0)	0.424

*Note*: The results are expressed as medians (IQR; interquartile ranges). The comparisons were calculated using the Mann–Whitney *U* test.

Abbreviations: HI, hyperactivity–impulsivity; IA, inattention.

### Study 3: Comparison by age group

As shown in Table 4, among the ADHD group, HI symptom scores were significantly lower in the 11–13 and 14–17 age groups compared to the 5–7 and 8–10 groups, while IA symptom scores did not differ significantly across age groups. Functional impairment scores for IA were significantly lower only in the 5–7 age group. In the non‐ADHD group, IA symptom scores were significantly lower in the 14–17 group compared to the 5–7 group, whereas HI symptom scores were significantly higher in the 5–7 group. No significant differences in functional impairment scores were observed in either group.

**Table 4:** Comparison of the symptom scores and impairment scores for both attention‐deficit/hyperactivity disorder (ADHD) and Non‐ADHD groups by age.

	I: aged 5–7	II: aged 8–10	III: aged 11–13	IV: aged 14–17	*p*‐value
ADHD	*n* = 51	*n* = 78	*n* = 71	*n* = 63	
IA symptom	13.0 (9.0–18.0)	14.5 (10.0–18.8)	14.0 (9.0–17.0)	12.0 (8.0–18.0)	0.476
IA impairment	4.0 (2.0–6.5)	6.5 (4.0–9.8)^†^	7.0 (5.0–10.0)^†^	7.0 (4.0–9.5)^†^	0.004
HI symptom	9.0 (6.5–14.5)	9.5 (6.0–14.0)	6.0 (3.5–9.0)^†,‡^	5.0 (1.0–9.0)^†,‡^	<0.001
HI impairment	4.0 (2.0–6.0)	6.0 (3.0–9.0)^†^	5.0 (1.5–9.0)	6.0 (2.0–9.0)	0.100
Non‐ADHD	*n* = 49	*n* = 44	*n* = 39	*n* = 57	
IA symptom	10.0 (7.0–15.0)	7.0 (5.0–11.0)	8.0 (2.5–11.5)	6.0 (3.0–10.0)^†^	0.004
IA impairment	3.0 (1.0–6.0)	3.0 (1.8–7.0)	5.0 (0.5–6.5)	3.0 (1.0–6.0)	0.572
HI symptom	6.0 (2.0–7.0)	2.5 (1.0–5.0)^†^	2.0 (0.5–5.0)^†^	1.0 (0–3.0)^†,‡^	<0.001
HI impairment	2.0 (1.0–5.0)	1.5 (0–6.0)	5.0 (0.5–6.5)	1.0 (0–4.0)	0.241

*Note*: The results are expressed as medians (IQR; interquartile ranges). The comparisons were calculated using the Kruskal–Wallis test with the Steel–Dwass method. ^†^ *p* < 0.05 (II, III, IV vs. I) and ^‡^ *p* < 0.05 (III, IV vs. II).

Abbreviations: HI, hyperactivity–impulsivity; IA, inattention.

### Study 4: Comparison by ADHD psychopharmacological treatment

Symptom scores did not differ significantly between participants with and without current pharmacotherapy; however, functional impairment scores were higher among medicated participants (Table 5), consistent with confounding by indication.

**Table 5:** Comparison of the symptom and impairment scores between presence or absence of the medication.

	With ADHD medication *n* = 130	Without ADHD medication *n* = 133	*p*‐value
IA			
Symptom score	14.0 (9.0–19.0)	13.0 (10.0–17.0)	0.27
Impairment score	7.0 (5.0–10.0)	5.0 (3.0–8.0)	<0.001*
HI			
Symptom score	8.0 (4.0–11.0)	7.0 (3.3–11.0)	0.436
Impairment score	6.0 (3.0–10.0)	4.0 (2.0–7.0)	<0.001*

*Note*: The results are expressed as medians (IQR; interquartile ranges). The comparisons were calculated using the Mann–Whitney *U* test. Asterisks indicate statistical significance (*p < 0.05).

Abbreviations: ADHD, attention‐deficit/hyperactivity disorder; HI, hyperactivity–impulsivity; IA, inattention.

## DISCUSSION

ADHD is a relatively prevalent psychiatric disorder that is often comorbid with various other mental disorders, such as depression and anxiety, particularly after adolescence. Accordingly, long‐term and continuous support is required. The ADHD‐RS is widely used for screening, severity assessment, and evaluating the effectiveness of therapeutic interventions. To the best of our knowledge, this cross‐sectional study is among the first in Japan to examine ADHD‐RS‐5 scores by diagnosis, age, and pharmacotherapy status. In particular, the relationship between pharmacological treatment and functional impairment scores on the ADHD‐RS‐5 has not been previously reported.

The proportion of younger children in this study was relatively high. This reflects the clinical characteristics of the Tokiwa Child Developmental Center, which places a strong emphasis on early intervention and routinely provides care for very young children. As a result, the patient population is younger than that typically seen in general child and adolescent psychiatry outpatient clinics. Many referrals originate from public health centers following developmental screenings conducted at the 1.5‐ and 3‐year health checkups in Sapporo City.

In Study 1, symptom scores for both IA and HI were significantly higher in the ADHD group across all age groups. Functional impairment scores were also generally higher in the ADHD group; however, there was no significant difference in HI scores within the 11–13 age group. It should be noted that 71.8% of children in the 11–13 non‐ADHD group were identified as exhibiting school refusal, which may have contributed to an overestimation of impairment unrelated to ADHD symptoms. Some trials and cohort studies indicate that functional impairment in ADHD is influenced by comorbidity and age, which may explain why certain age bands did not show significant differences in HI‐related impairment in our sample.^12^, ^13^

In this study, 60% of children diagnosed with ADHD also had ASD. Previous studies have reported comorbidity rates of approximately 40%–70%, and the results of this study are consistent with those findings.^14^, ^15^, ^16^, ^17^, ^18^ Furthermore, while ADHD prevalence has been reported to decrease with age, the current study population included many younger children, primarily in early childhood. Zablotsky et al. reported that the group with comorbid ASD had higher treatment needs and more comorbid conditions than the ADHD‐only group.^19^ The results of this study are from a single‐center study with a limited number of cases. Therefore, increasing the sample size could potentially yield statistically significant differences. These findings suggest that the impact of ASD comorbidity may not be fully captured by ADHD symptom severity alone, highlighting the importance of evaluating ASD‐specific characteristics in future studies.

In the ADHD group, HI symptom scores were significantly lower in the 11–13 and 14–17 age groups. Previous studies have reported that HI tends to decline with age, and our findings are consistent with this notion.^13^, ^20^, ^21^ In contrast, IA symptom scores did not significantly differ across age groups, while functional impairment scores were significantly lower in the 5–7 age group. This may suggest that inattentive symptoms are present at a similar level across ages, but are less likely to be perceived as problematic in younger children due to lower social demands. Previous studies have indicated that IA symptoms tend to remain relatively stable over time, and no conclusive evidence has been identified to suggest systematic progression or worsening of these symptoms.^22^ These findings emphasize the clinical importance of systematically assessing inattentive symptoms, even in younger children suspected of having ADHD.

In this study, children with ADHD who were receiving medication exhibited significantly higher functional impairment scores for both IA and HI compared to those not receiving ADHD medication. Both Japanese and international clinical guidelines recommend psychosocial interventions as the first‐line treatment for ADHD, with pharmacotherapy considered for moderate to severe cases when necessary.^23^, ^24^, ^25^, ^26^ Notably, the American Academy of Pediatrics emphasizes the importance of shared decision‐making with caregivers when initiating pharmacological treatment.^27^

Functional impairment scores on the ADHD‐RS‐5 may index caregiver‐perceived burden and could inform clinical conversations about treatment, while requiring validation in adjusted models and longitudinal cohorts. Although symptom scores did not differ significantly by psychopharmacological treatment status, this finding may reflect the therapeutic effects of medication.

This study has several limitations. First, because teacher ratings were not collected, it was not possible to assess symptoms across multiple settings. As a result, the study does not offer a comprehensive diagnostic evaluation that incorporates input from both parents and teachers. Second, the identities of the raters were not specified. Although most were mothers, some respondents may have been fathers, grandparents, or facility staff, introducing potential rater bias. Third, the non‐ADHD group consisted of children referred to a child psychiatry outpatient clinic and, therefore, may not be representative of the general population. Fourth, detailed information regarding medication therapy was not collected. Some children may have recently started ADHD medication, while others may have been undergoing long‐term treatment or changes in their medication regimen. Many cases were already receiving ADHD or antipsychotic treatment at the time of referral. Due to potential case number biases based on the type of ADHD medication used, analysis of the specifics of ADHD medication, treatment duration, and combination therapy remains a future task. Therefore, these findings cannot be used to establish definitive criteria for initiating medication therapy. Fifth, adjustments for potential confounding factors, such as gender distribution, the time interval between initial diagnosis and the study period, and the presence of concomitant use of other psychotropic medications, were insufficient. Further research using multivariate models is needed. Finally, as this is a cross‐sectional study, comparisons between age groups reflect differences between cohorts rather than within‐individual changes over time. Therefore, utmost caution is required to ensure that these results are not interpreted as reflecting within‐individual symptom changes attributable to aging.

## CONCLUSION

The results confirmed that children with ADHD exhibit higher symptom and functional impairment scores compared to those without ADHD, supporting its construct validity in this setting. Additionally, HI symptoms were found to decline with age, whereas inattentive symptoms remained stable, underscoring the importance of early and continuous assessment of IA, even in younger children. Furthermore, functional impairment scores were significantly higher among children receiving medication, suggesting the scale's potential role in assessing severity and informing treatment decisions.

## AUTHOR CONTRIBUTIONS

Ryotaro Shimomura drafted the article and prepared the figures and tables. Ryotaro Shimomura, Keisuke Oyatani, and Masaru Tateno developed the conception and design of the study. Masaru Tateno edited the main text and supervised this study. Ryotaro Shimomura, Yukie Tateno, Kotaro Nanba, Eri Shiraishi, and Masaru Tateno corrected the data. Ryotaro Shimomura and Keisuke Oyatani analyzed and interpreted the data. All authors read and approved the final manuscript.

## CONFLICT OF INTEREST STATEMENT

The authors declare no conflicts of interest.

## ETHICS APPROVAL STATEMENT

The Ethics Committee of Tokiwa Hospital approved this study. Informed consent was obtained from all caregivers and, when appropriate, assent from children. This study was conducted in accordance with the Declaration of Helsinki guidelines.

## PATIENT CONSENT STATEMENT

N/A.

## CLINICAL TRIAL REGISTRATION

N/A.

## Data Availability

The data that support the findings of this study are available on request from the corresponding author. The data are not publicly available due to privacy or ethical restrictions.
